# Characterization of the Raf Kinase Inhibitory Protein (RKIP) Binding Pocket: NMR-Based Screening Identifies Small-Molecule Ligands

**DOI:** 10.1371/journal.pone.0010479

**Published:** 2010-05-05

**Authors:** Anne N. Shemon, Gary L. Heil, Alexey E. Granovsky, Mathew M. Clark, Dan McElheny, Alexander Chimon, Marsha R. Rosner, Shohei Koide

**Affiliations:** 1 Ben May Department for Cancer Research, University of Chicago, Chicago, Illinois, United States of America; 2 Department of Neurobiology, Pharmacology and Physiology, University of Chicago, Chicago, Illinois, United States of America; 3 Department of Biochemistry and Molecular Biology, University of Chicago, Chicago, Illinois, United States of America; Auburn University, United States of America

## Abstract

**Background:**

Raf kinase inhibitory protein (RKIP), also known as phoshaptidylethanolamine binding protein (PEBP), has been shown to inhibit Raf and thereby negatively regulate growth factor signaling by the Raf/MAP kinase pathway. RKIP has also been shown to suppress metastasis. We have previously demonstrated that RKIP/Raf interaction is regulated by two mechanisms: phosphorylation of RKIP at Ser-153, and occupation of RKIP's conserved ligand binding domain with a phospholipid (2-dihexanoyl-sn-glycero-3-phosphoethanolamine; DHPE). In addition to phospholipids, other ligands have been reported to bind this domain; however their binding properties remain uncharacterized.

**Methods/Findings:**

In this study, we used high-resolution heteronuclear NMR spectroscopy to screen a chemical library and assay a number of potential RKIP ligands for binding to the protein. Surprisingly, many compounds previously postulated as RKIP ligands showed no detectable binding in near-physiological solution conditions even at millimolar concentrations. In contrast, we found three novel ligands for RKIP that specifically bind to the RKIP pocket. Interestingly, unlike the phospholipid, DHPE, these newly identified ligands did not affect RKIP binding to Raf-1 or RKIP phosphorylation. One out of the three ligands displayed off target biological effects, impairing EGF-induced MAPK and metabolic activity.

**Conclusions/Significance:**

This work defines the binding properties of RKIP ligands under near physiological conditions, establishing RKIP's affinity for hydrophobic ligands and the importance of bulky aliphatic chains for inhibiting its function. The common structural elements of these compounds defines a minimal requirement for RKIP binding and thus they can be used as lead compounds for future design of RKIP ligands with therapeutic potential.

## Introduction

The Raf-1 kinase inhibitory protein (RKIP) serves as a regulator of the MAP kinase signal transduction cascade through its interaction and inhibition of Raf-1 kinase activity [1], [2]. Phosphorylation of RKIP at Serine-153 by protein kinase C abolishes RKIP's inhibition of Raf-1 [3] and converts it to an inhibitor of the G-protein coupled receptor kinase, GRK-2, thus facilitating cross-talk between the EGF and GPCR signaling pathways [4]. Furthermore, RKIP functions as a metastatic tumor suppressor in prostate [5] and breast cancers [6], sensitizes human prostate and breast cancer cells to drug induced apoptosis and regulates the integrity of the cell cycle via the spindle checkpoint [7], [8] (reviewed in [9]). These reports highlight the significance of RKIP in mammalian cells as a vital regulator of mitosis, cell differentiation and apoptosis.

Long before its function as a kinase inhibitor was identified, RKIP was isolated from the soluble fraction of bovine brain extracts through affinity chromatography to an immobilized dye (Cibacron Blue) [10]. This dye is generally considered a nucleotide analog, and thus the affinity of RKIP to the dye suggested a nucleotide-binding function for RKIP. In addition, gel filtration and lipid affinity chromatography were used to demonstrate binding of a number of organic anions, steroids and also the lipid membrane component, phosphatidylethanolamine [10], [11]. Consequently, RKIP was originally named PEBP for its function as a phosphatidylethanolamine binding protein. Subsequent studies demonstrated that RKIP binds only negatively charged phosphatidylglycerol in liposomes and lipid monolayers [12]. The macroscopic methods used in these previous studies did not identify the specific binding site(s) for these interactions nor determine site-specific affinity of these ligands to RKIP. Also the effective concentrations of ligands in these methods are very high, and consequently they can detect weak binding events that may not be physiologically relevant.

Amino acid sequence analysis of RKIP has identified it as a member of a large protein family named the PEBP family. This family has homologs present from bacteria to mammals [13], [14], [15], [16]. Among the mammalian members of this family, the pocket region between residues 63 and 126 (based on the bovine RKIP numbering scheme [17]) displays a particularly high level of sequence identity [18], suggesting a conserved function for this region. The structures of RKIP and its homologs from a number of species have been determined using x-ray crystallography [17], [19], [20], [21], [22], [23]. These crystal structures reveal a globular protein with a beta-sheet core and a prominent solvent exposed pocket. The residues that form the pocket are among the aforementioned highly conserved residues. In three of the five x-ray structures of mammalian RKIP proteins a small ionic ligand is bound in this pocket [17], [19], confirming this pocket as a ligand-binding site. While two of these three structures (Protein Data Bank entries 1A44 and 1BEH) contain a small ion from the buffer as the ligand, the third (PDB id 1B7A) contains O-phosphorylethanolamine (PE), the soluble polar head group of the membrane phospholipid phosphatidylethanolamine. The RKIP-PE complex is the only crystal-based structural evidence to date for the binding of an organic ion to the pocket. Although PE is a phospholipid head group and the crystal structure may represent the mode of phospholipid–RKIP interaction, this interpretation is still speculative because PE lacks the diacylglycerol moiety that constitutes the majority of the phospholipids. Moreover, the crystals of RKIP with PE bound were formed at pH 4.6 in the presence of 100 mM PE [17], and the other crystal structures of mammalian RKIP with ligands bound to the pocket were also determined at low pH (≤ pH 5.5). Thus, little is known about the mode(s) of RKIP-ligand interactions under near physiological solution conditions.

Although the previous studies have reported a number of RKIP ligands, they collectively lack definitive data regarding binding affinity, the location of the binding site(s) as well as the ligand specificity of RKIP in solution under near physiological conditions. The chemical diversity of the previously identified ligands raises fundamental questions about the structural basis for RKIP–ligand interactions: Does the pocket have an extremely broad binding specificity so that it can bind to all of these ligands? If so, what is the chemical space for the pocket ligand? Or alternatively, does RKIP have another binding site distinct from the pocket? To address these questions it is necessary to investigate ligand binding by a method that permits quantitative and structural analysis of protein-ligand interactions under near physiological solution conditions.

Solution NMR spectroscopy meets the above criteria for high-resolution and quantitative analysis of protein-ligand interactions. Ligand binding experiments using NMR spectroscopy require relatively low protein concentrations and thus allow for flexibility in the choice of solution conditions. In addition, it has the ability to detect weak interactions and to map specific protein residues affected by ligand binding. These attributes make NMR spectroscopy an ideal tool to perform a comprehensive analysis of the ligand binding properties of RKIP, which was not possible with the previously implemented techniques. Furthermore, it can also determine the site-specific affinity of the interactions [24] providing a significant advantage over the methods previously used to study RKIP-ligand interactions. We have reported the sequence specific resonance assignments of RKIP at pH 7.4 [25], the prerequisite for NMR-based structural analysis [26], providing the foundation for detailed characterization of RKIP-ligand interactions by NMR spectroscopy. Recently, we characterized the binding of locostatin, a putative RKIP inhibitor, to the RKIP pocket [27].

In this study, we used NMR-based screening to assay interactions of RKIP with a number of potential ligands by NMR spectroscopy. Surprisingly, the binding of many compounds previously postulated or reported to interact with RKIP was not detectable at near physiological solution conditions even at millimolar concentrations of the compounds. In contrast, we discover three novel ligands for RKIP, and we provide evidence for site-specific binding of these compounds to the pocket and their binding affinity. In addition, we examined the biological effect of these compounds and identified one that had off target effects which reduced MAPK activity. Taken together, this work defines the ligand-binding properties of RKIP and suggest its potential role as a scavenger for hydrophobic compounds within cells.

## Results

### NMR assay of ligand binding to RKIP

To investigate whether potential non-lipid ligands reported in the literature actually bind to RKIP and, if so, to define their binding site, we tested the interactions between RKIP and these compounds using NMR spectroscopy [21], [25]. By comparing the ^1^H,^15^N-heteronuclear single-quantum correlation (HSQC) spectra of ^15^N-labeled rat RKIP (rRKIP) in the presence and absence of a potential ligand, we can detect ligand binding as perturbations to the NMR cross peaks (referred to as “chemical shift perturbations” hereafter) [24]. Note that only the backbone and side chain amide moieties of the ^15^N-rRKIP give rise to NMR signals while an unlabeled ligand is spectroscopically silent. The recently completed sequence-specific NMR assignments for rRKIP [25] allow us to identify the residues affected by ligand binding and thus to map the binding site.

**Figure 1:**
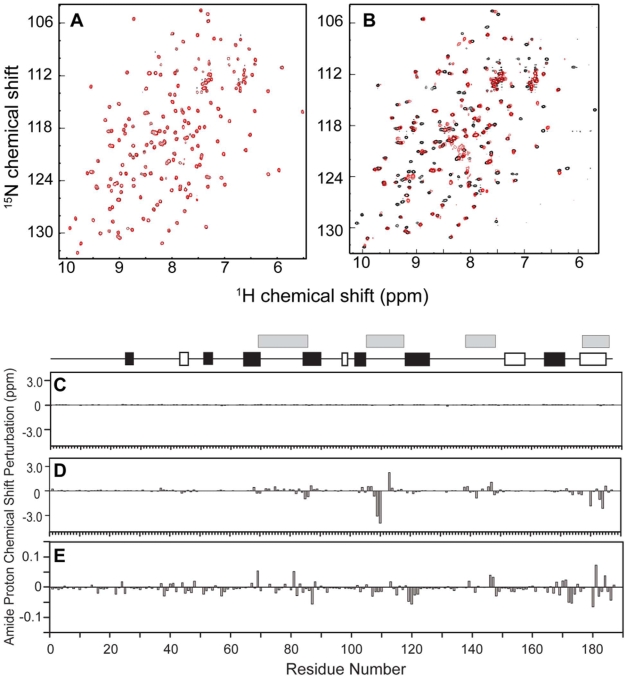
Overlay of ^1^H,^15^N-HSQC spectra of RKIP in the absence (black) and presence (red) of a potential ligand. (**A**) PE (10 mM) does not result in any significant chemical shift perturbations. (**B**) ^1^H,^15^N-HSQC spectra of RKIP taken at pH 6.0 (red) and at pH 7.4 (black) showing a large number of missing peaks at pH 6.0. In all the panels, red contours are plotted over black contours, and thus a black cross peak appears only when its corresponding peak drawn in red is either shifted or missing. RKIP chemical shift perturbations for amide proton resonances by PE (10 mM) (C) or DHPE (4.5 mM) (D) at pH 7.4 plotted as a function of residue number. Secondary structural elements are depicted at the top with beta-sheet and alpha-helices represented by closed and open boxes, respectively. Gray boxes indicate the position of residues that form the ligand-binding pocket. (**E**) RKIP chemical shift perturbations by PE (100 mM) at pH 6.0. Note that panel E is plotted on a smaller vertical scale than that for C and D.

Non-lipid molecules assayed for binding included: O-phosphorylethanolamine (PE), the head group of phosphatidylethanolamine; the nucleotides GDP, GTP and cAMP; the phosphorylated amino acids O-phosphorylserine, O-phosphorylthreonine and O-phosphoryltyrosine; tryptophan, phenylalanine and inorganic phosphate (P*_i_*) (**Table S1**). Surprisingly, under the near physiological solution conditions used (pH 7.4, 100 mM NaCl, 30°C), none of these non-lipid compounds caused significant chemical shift perturbations for the rRKIP residues, even at very high ligand concentrations used (**Table S1** and **Figure S1**). This suggests the importance of examining ligand binding to RKIP in solution under near-physiological conditions. In contrast, a previously identified RKIP ligand, DHPE, caused large perturbations (**Figure 1D**). Interestingly, although PE had previously been co-crystallized with bovine RKIP at low pH [17], it did not interact with the protein under these more physiological conditions (**Figure 1A** and **C**). Together, these results indicate that rRKIP has very weak affinity, if any, to these compounds.

### Effects of pH on RKIP structure

An apparent conundrum in this study is the lack of binding of PE to rat RKIP in solution despite the successful crystallization of the bovine RKIP/PE complex [17]. Because the rat and bovine proteins are highly homologous, particularly among the residues forming the pocket, it is unlikely that these proteins have distinctly different ligand-binding properties. The RKIP/PE complex was crystallized at pH 4.6, which is much lower than the pH used in our solution studies (pH 7.4). The pocket contains a conserved His residue (His-85 in the bovine RKIP numbering), and the pKa of His is typically between 6.0-7.0 [28]. These observations led us to hypothesize that pH may affect the structure and function of the RKIP pocket. To investigate the effect of pH, we performed pH titration of RKIP monitoring the amide proton chemical shifts with the ^15^N-HSQC spectrum.

As pH was titrated below pH 6.0, a large number of peaks exhibited a dramatic loss in peak intensity (**Figure 1B**), characteristic of an increase in motion on the μs-ms timescale [26]. These peaks were mapped to residues in the loops that form the binding pocket. Because of the peak intensity decrease, we could not accurately determine the pKa values for these residues, which prevented us from identifying the origin of the pH-induced structural perturbations in the pocket region. Nevertheless, these results clearly indicate that the pocket conformation and/or dynamics are sensitive to pH change and that these loop regions are able to sample multiple conformations while rRKIP retains the overall, folded conformation.

There were other HSQC peaks that exhibited significant pH-dependent migration but no significant decrease in intensity. We were able to determine the pKa value for 46 residues of this class. We did not find a clear clustering pattern in the distribution of residues with similar pKa values, except for two discrete sites distal to the conserved pocket corresponding to regions adjacent to solvent exposed histidines at positions 23 and 159 (data not shown). Moreover, the residues near His-23 and -159 do not exhibit ligand-induced chemical shift perturbations at pH 7.4, suggesting that these sites and the pocket are structurally independent and pH-dependent structural changes at these sites do not affect ligand binding to the conserved pocket.

### Effects of pH on ligand binding by RKIP

The above results suggest that an increased flexibility at lower pH might alter the ligand-binding properties of RKIP. To investigate the effect of lower pH on ligand binding, a number of ligands tested at pH 7.4 were reexamined for binding to RKIP at pH 6.0. This pH value was chosen because below this pH value we observed a significant loss of spectral quality. In the presence of PE and O-phosphoryltyrosine at pH 6.0, a number of residues in the loops surrounding the ligand pocket of RKIP displayed significant broadening of amide proton peaks in the HSQC spectra. However, chemical shift perturbations for pocket residues were very small, only slightly above background in these spectra (**Figure 1E**), much smaller than those observed for phospholipid compounds (**Figure 1D**). These results suggest that PE and O-phosphoryltyrosine have very low affinity for RKIP at this pH. The phosphate group of PE has a reported pKa of ∼5.8, suggesting that the affinity of PE may be much higher at lower pH than at 6.0. However, due to the limitations discussed above, we were not able to investigate this possibility. Our data suggests that the presence of PE in the crystal structure of bovine RKIP (PDB id: 1b7a) may be a product of the weak affinity of PE at low pH coupled with its high concentrations used during crystallization.

**Figure 2:**
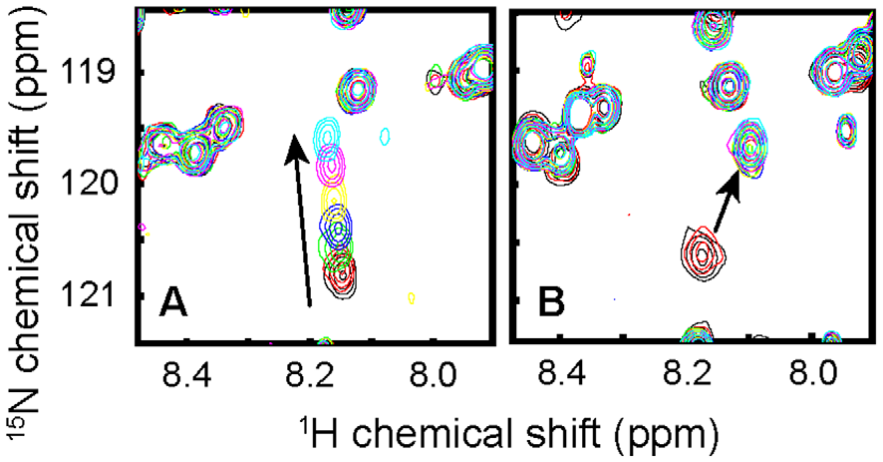
A portion of RKIP HSQC spectra illustrating the difference in the DHPA-induced chemical shift perturbations of residue 182 at pH 7.4 and 6.0. (**A**) DHPA titration at pH 7.4 causes the peak to continuously shift, characteristic of fast exchange kinetics. (**B**) Titration at pH 6.0 causes the peak intensity to decrease with a concomitant appearance of a new peak, characteristic of slow exchange kinetics. The arrows indicate the trajectories of cross peak movements. Panel (**A**) shows data with DHPA concentrations of 0, 0.05 0.25, 0.5, 1.0, 2.5 and 7.5 mM, and (**B**) data with 0, 0.05, 0.25, 0.5, 1.0, 2.5 and 4.5 mM DHPA.

Two of the four compounds exhibiting binding at pH 7.4, DHPG and DHPS, demonstrated similar patterns of chemical shift perturbations at pH 6.0 as previously described [21]. Titration of the phospholipids at pH 6.0 confirmed interactions with similar affinity for DHPG and DHPS to that observed at pH 7.4 (**Table S1**). In contrast, while PA binding at pH 6.0 perturbed a similar subset of residues as it did at pH 7.4, the trajectory of the peak shifts was different. As PA was titrated in, the HSQC peaks did not change their positions but their intensities decreased, and a new set of peaks appeared (**Figure 2A**). This behavior is characteristic of slow interconversion between the free and ligand-bound states [26]. Furthermore, the affinity of PA for RKIP was at least 3-fold higher at pH 6.0 than at pH 7.4 (**Figure 2B**). The mono-substituted phosphate moiety of PA has a pKa of 8.0 [29] indicating that its protonation state is sensitive to changes in pH between pH 7.4 and pH 6.0. In comparison, the pKa values of the other phospholipids examined in this study are expected to be much lower, as the pKa of the phosphate group of dimyristoyl-phosphatidylethanolamine has been calculated to be ∼1.7 [29]. The remarkable similarities between the peak shift patterns observed for DHPE, DHPG and DHPS at pH 6.0 and pH 7.4 are in stark contrast with the differences seen for PA binding at the two pH values, suggesting that the higher affinity for PA at pH 6.0 results from the protonation of the ligand and not from a pH-dependent effect on the RKIP binding pocket itself. Together, these results suggest that the compounds that showed no significant binding in our NMR assays (**Table S1**) have very weak affinity at best to RKIP in near physiological pH range.

### NMR-based chemical library screen reveals three novel RKIP ligands

The failure of previously reported compounds, except for the short-chain lipid, to bind RKIP at physiological pH has revised our understanding of RKIP's ligand binding properties. RKIP is not an extremely promiscuous binding protein. Therefore, in order to better define the chemical space of RKIP ligands and also to discover lead compounds for RKIP inhibitors, we have performed NMR-based screening. We utilized the chemical shift perturbation method described above and screened approximately 6, 000 compounds from a commercially available library of “drug-like” small organic chemical compounds.

**Figure 3:**
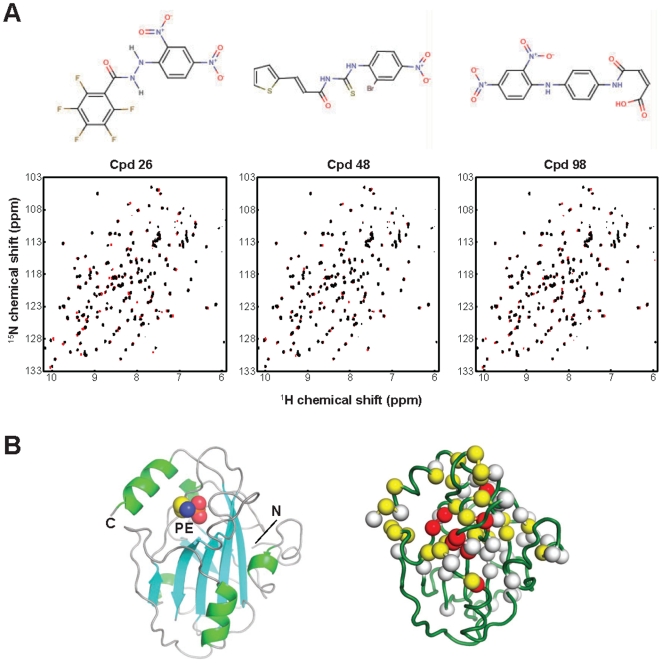
Newly identified RKIP ligands. (**A**) The chemical structures of compounds **26**, **48** and **98** and their effects on the RKIP HSQC NMR spectrum. The ^1^H,^15^N HSQC spectrum of RKIP taken in the presence of the indicated compound is plotted in red and a control spectrum in the absence of a compound in black. The black spectrum is plotted over the red one so that red cross peaks are visible only for those that are perturbed by the compound (B) A cartoon representation of the crystal structure of RKIP bound to PE (left; PDB id, 2IQX), and the locations of residues whose HSQC peaks were perturbed by compound **26**. Red, yellow and white spheres indicate the Cα positions for residues whose peaks were shifted by more than two peak widths, 1 to 2 peak withs and less than one peak width, respectively. Because the three compounds perturbed very similar sets of residues, only data for compound **26** are shown.

We have identified three compounds: N'-(2,4-dinitrophenyl)-2,3,4,5,6-pentafluorobenzohydrazide (compound **26**), N-{[(2-bromo-4-nitrophenyl)amino]carbonothioyl}-3-(2-thienyl)acrylamide (compound **48**) and 4-({4-[(2,4-dinitrophenyl)amino]phenyl}amino)-4-oxo-2-butenoic acid (compound **98**) (**Figure 3A**). These compounds caused unambiguous NMR chemical shift perturbation (**Figure 3A**). The three compounds affected very similar sets of cross peaks, indicating their similar modes of binding. Most of the HSQC peaks affected by these compounds were mapped to residues surrounding the pocket (**Figure 3B**), indicating that the pocket is a preferred binding site for RKIP ligands and RKIP does bind to small organic compounds that are chemically distinct from lipids (**Figure 3B**). The three compounds include either 2,4-dinitrophynyl amine or 2-bromo-4-nitrophynyl amine moieties (**Figure 3A**), suggesting that the 4-nitrophenyl group serves as a common element recognized by RKIP. Among the screened compounds, there were a total of 19 others containing either 2,4-dinitrophynyl amine or 2-bromo-4-nitrophynyl amine moieties that exhibited no sign of RKIP binding, suggesting that the substituted nitrophenyl amine groups are not sufficient for RKIP binding.

### Newly identified compounds have no effect on RKIP-Raf-1 interaction and RKIP phosphorylation

**Figure 4:**
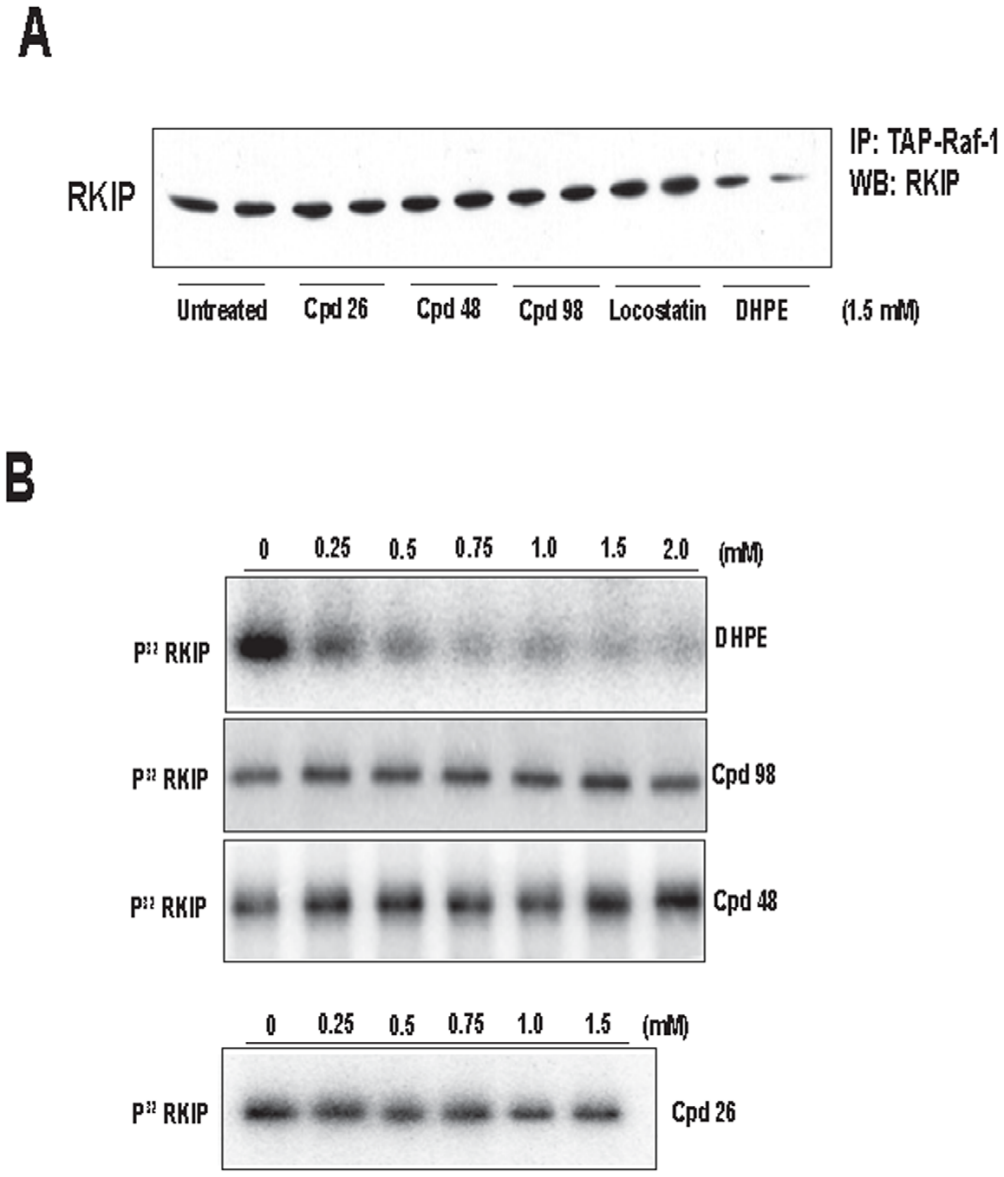
DHPE but not compounds 98, 48, 26 nor locostatin decreases RKIP interactions with Raf-1 and PKC. (**A**) Representative western blot of RKIP association with Raf-1 in the presence of 1.5 mM Compounds **26**, **48** or **98** as well as locostatin or DHPE. (**B**) PKC phosphorylation of RKIP in the presence of increasing concentrations of DHPE or compounds **98**, **48**, or **26** (Representative autoradiogram is shown).

In our previous studies, we have shown that RKIP pocket occupancy by the short-chain lipid, DHPE, causes dissociation of RKIP from Raf-1 [21]. In contrast, we observed that locostatin, which also bound to the RKIP pocket, had no effect on RKIP-Raf-1 interaction [27]; we have attributed locostatin's inability to perturb RKIP function to its smaller size than DHPE. The size of the three identified compounds is similar to that of locostatin and also had no effect on Raf-1 interaction (**Figure 4A**). Furthermore, we monitored RKIP phosphorylation by PKC in the presence of the three compounds and used DHPE as a control. In the presence of DHPE, as previously shown, RKIP phosphorylation was reduced in a dose dependent manner (**Figure 4B**). By contrast, as observed with locostatin, increasing concentrations of these compounds (up to 2.5 mM) had no effect on RKIP phosphorylation by PKC (**Figure 4B**).

### Compound 48 impairs EGF-induced MAPK in HeLa cells

**Figure 5:**
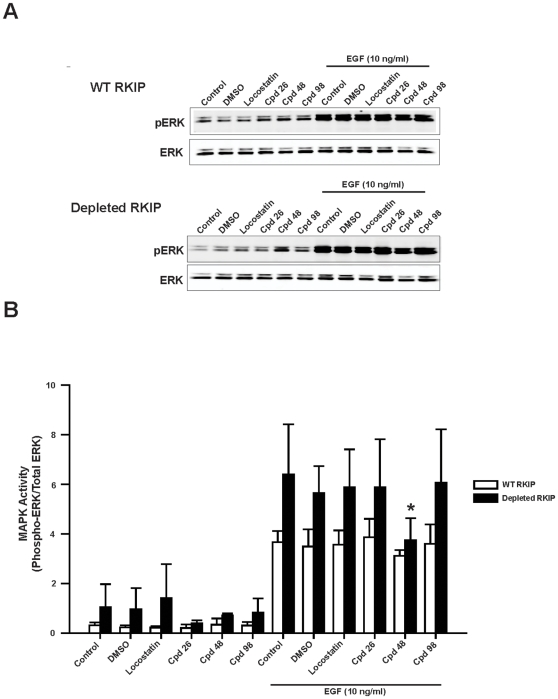
Compound 48 reduces EGF-induced MAPK activity. HeLa cells expressing wild-type or depleted RKIP were serum starved overnight and then pre-treated for 30 min with 20 µM compounds (as indicated). EGF (10 ng/ml) was added in the final 5 minutes of incubation. (**A**) Whole cell lysates (30 µg) were separated on SDS-PAGE gels (12.5%), transferred to nitrocellulose and analysed by immunoblotting with anti-phospho ERK (*Top panel*: representative Western blot) and anti-total ERK antibodies (*Bottom panel*: representative Western blot). (**B**) ERK phosphorylation was assessed by normalizing phospho-ERK levels to total ERK levels as depicted in bar graphs (Mean ± SEM; n = 3 independent experiments). ^*^p<0.05 indicates the significance of change relative to the DMSO control in the presence of EGF.

**Figure 6:**
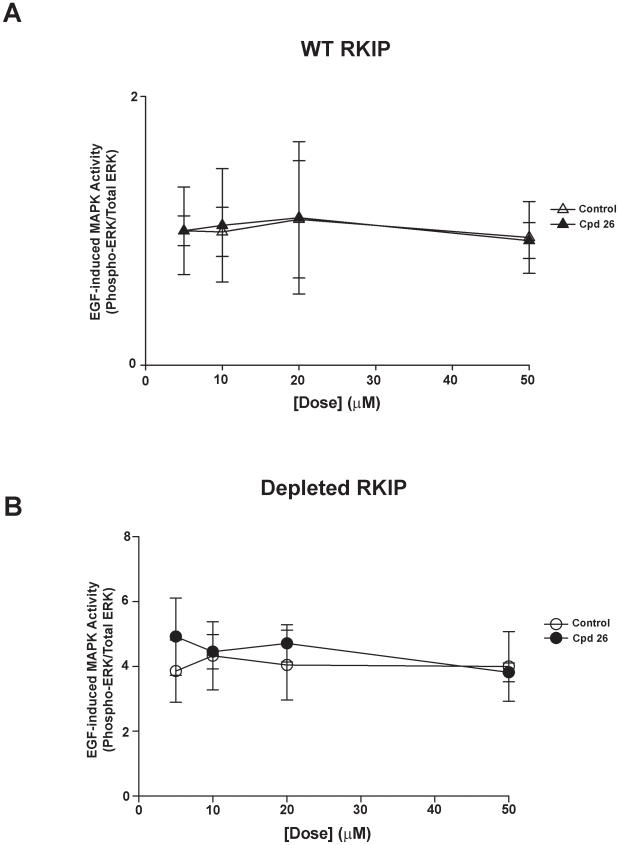
Increasing doses of compound 26 does not change EGF-induced MAPK activity. HeLa cells expressing wild-type (**A**) or depleted RKIP (**B**) were serum starved overnight and then pre-treated for 30 min with increasing doses of DMSO (control) or Compound **26**. EGF (10 ng/ml) was added in the final 5 minutes of incubation. Whole cell lysates (30 µg) were separated on SDS-PAGE gels (12.5%), transferred to nitrocellulose and analysed by immunoblotting with anti-phospho ERK and anti-total ERK antibodies. ERK phosphorylation was assessed by normalizing phospho-ERK levels to total ERK levels as depicted in line graphs. The results shown are mean ± range of two independent experiments.

**Figure 7:**
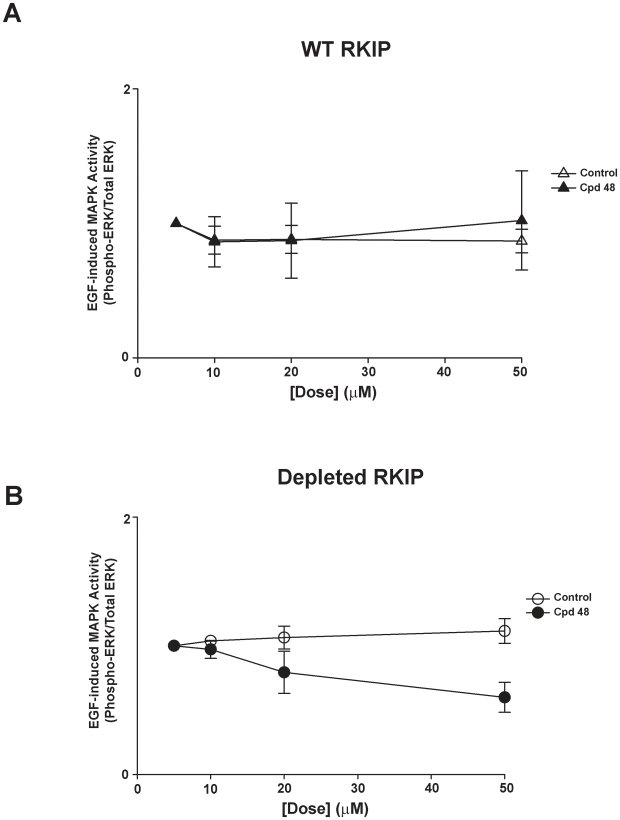
Compound 48 reduces EGF-induced MAPK activity dose dependently. HeLa cells (0.5×10^6^ cells/ml) expressing wild-type (**A**) or depleted RKIP (**B**) were serum starved overnight and then pre-treated for 30 min with increasing doses of DMSO (control) or compound **48**. EGF (10 ng/ml) was added in the final 5 minutes of incubation. Whole cell lysates (30 µg) were separated on SDS-PAGE gels (12.5%), transferred to nitrocellulose and analysed by immunoblotting with anti-phospho ERK and anti-total ERK antibodies. ERK phosphorylation was assessed by normalizing phospho-ERK levels to total ERK levels as depicted in line graphs. The results shown are mean ± range of two independent experiments.

We have previously shown that EGF-induced MAPK activity is augmented in HeLa cells depleted of RKIP [1]. If RKIP binding to Raf-1 requires pocket binding, then compounds that bind the RKIP pocket would interfere with downstream signaling to MAPK. However, we showed that locostatin binds to the RKIP pocket with no effect on Raf-1 association nor RKIP phosphorylation by PKC and did not change the EGF-induced MAPK activity in HeLa cells in the presence or absence of RKIP [27]. Similarly, two of the compounds (**98** and **26**) from our screen mimicked the lack of effect by locostatin on EGF-induced MAPK activity (**Figure 5**). In addition, we observed compound **48** at 20 µM had no effect on wild-type HeLa cells; however compound **48** reduced MAPK activity by 34±11% (mean ± SEM, n = 3) in RKIP depleted cells (**Figure 5**). To further examine the effect of compound **48**, we compared the dose response of compound **26** as a control to that of compound **48** (**Figure 6** and **7**). As predicted, compound **26** at all concentrations tested had no effect on EGF-induced MAPK activity in HeLa cells in the presence or absence of RKIP (**Figure 6**). In contrast, the inhibitory effect was dose-dependent for compound **48** with respect to RKIP depleted cells but dose-independent in wild-type RKIP cells (compare **Figure** **7A** with **7B**).

### Compound 48 reduced cell viability in HeLa cells

**Figure 8:**
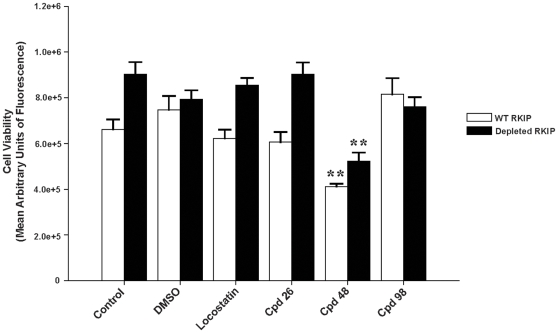
Compound 48 reduces cell viability in HeLa cells. HeLa cells (4×10^4^ cells/ml) expressing wild-type (**open bars**) or depleted RKIP (**solid bars**) were serum starved overnight and then pre-treated for 30 min with 20 µM DMSO (control) or compound **48**. Reaction was stopped by the addition of cell titer blue (20 µl) to each well and incubated for 2 h at 37°C. Metabolic activity was measured on a fluorometer (Fluorescence 560_Ex_/590_Em_) and the results are described as the mean arbitrary units of fluorescence of triplicate wells of two independent experiments. ^**^p<0.01 indicates the significance of the change relative to the corresponding sample in the absence of compound **48**.

To examine the effect of these compounds on HeLa cells, we assayed cell viability as assessed by metabolic activity in the presence or absence of the compounds from the screen. All compounds examined with the exception of compound **48** had no effect on cell viability. In contrast, compound **48** reduced cell viability by 43±6 and 34±6% in wild-type and RKIP depleted cells respectively (mean ± SEM; n = 4; **Figure 8**). In contrast to the viability assays, MAP kinase assays are assessed after shorter exposure to the drug which may account for the differential response to compound **48** between RKIP-expressing and RKIP-depleted cells observed in the MAP kinase assay. Taken together, these results suggest that compound **48** is toxic at 20 µM and may result in off target effects.

## Discussion

Despite a number of investigations into the ligand binding properties of RKIP, there has remained a lack of detailed quantitative and structural analysis of ligand binding in solution. In this study, we used NMR spectroscopy to address a number of fundamental questions raised by the diversity of ligands reported to bind RKIP and to provide definitive quantitative structural data regarding the ligand specificity of RKIP. Through this effort we provide the first comprehensive structural analysis of ligand binding to the conserved pocket of RKIP in solution.

We also found significant pH-dependent changes of HSQC peak intensity for residues surrounding the pocket. Although the loss in peak intensity precluded detailed analysis, these results are indicative of changes in the conformation and/or dynamic properties of the region at lower pH. We found evidence for weak binding of PE at pH 6.0. Likewise, the affinity of PA was higher at pH 6.0 relative to that at pH 7.4. However, lowered pH does not alter the binding of DHPE, DHPG and DHPS, strongly suggesting that the pocket is unaffected by pH change over this range. The difference in the binding affinity of PA between the two pH values can be explained by a change in the protonation state of the phosphate group of PA, suggesting that the microenvironment of the cell will determine the localized effect of ligand binding as well as protein-protein interaction. RKIP binds more strongly to the di-anionic form than the mono-anionic form of the phosphate group. It is, however, possible that a further decrease in pH, particularly in the range near the pKa of carboxylates (4–4.5) may induce substantial changes in the RKIP pocket structure and its ligand-binding properties. Also in this study, we observed that there are conserved His residues that are sensitive to pH which suggests that they may be involved in localized protein-protein interaction and are not involved in ligand binding.

While prior structural information regarding binding of ligands to the conserved pocket of RKIP was limited to crystal structures that contained only small anions [17], [19], this work significantly expands our knowledge of the chemical space for RKIP ligands and demonstrates a definitive specificity for phospholipids under near physiological conditions. The binding analysis of a drug-like compound, locostatin [30], its precursors and their stereoisomers revealed that the RKIP pocket can also bind to non-lipid compounds with a certain level of stereospecificity [27]. The RKIP ligands identified in this work all bound to the conserved pocket, and no other binding sites were found. These observations strongly suggest that the pocket is the main, if not the only, binding site in RKIP for organic compounds.

These initial studies lead to screening for compounds that had a similar structure as the non-lipids described in **Table S1**. Essentially these were planar hydrophobic compounds that, unlike locostatin, were not capable of chemical cross-linking, which made them easily studied by NMR. These compounds bound to the pocket with an affinity similar to locostatin; however, their side chains allowed for binding to different regions within the RKIP binding pocket.

One motivation in this screen was to identify a compound that affected RKIP function via pocket binding. From this study we established that these compounds, even up to millimolar concentrations did not affect RKIP association with Raf-1 and did not alter Ser-153 phosphorylation by PKC. These results confirm and extend previous findings that locostatin can similarly bind the RKIP pocket without altering association with Raf-1 or kinases such as PKC. Based on these findings, we postulated that MAPK should not be affected by drug binding. However, one out of the three compounds (compound **48**) displayed off target effects by reducing MAPK activity as well as metabolic activity in HeLa cells. This effect was more pronounced in RKIP depleted cells suggesting a transient protective role for RKIP with respect to MAPK activation, against toxic drugs as previously observed for locostatin.

In this study, we describe the importance of the microenvironment with respect to pH for ligand binding to the RKIP pocket. The observation that bulky lipids such as DHPE inhibit both Raf-1 and kinase association with RKIP suggests that interaction with residues in the vicinity of the pocket rather than binding within the pocket is required for these protein-protein interactions. We also identified three compounds that bind to the RKIP binding pocket with no effect on Raf-1 association or RKIP phosphorylation. One out of the three compounds from this screen (compound **48**) reduced MAPK activation in a dose dependent manner independent of RKIP, suggesting off target effects. The structure of these compounds which bind to RKIP should act as lead compounds for future drug design which could ultimately lead to intervention for diseases that develop from disregulation of the pathways involving RKIP and/or its targets.

## Materials and Methods

### Reagents and antibodies

A compound library containing 10, 000 drug-like chemicals was purchased from ChemBridge. N'-(2,4-dinitrophenyl)-2,3,4,5,6-pentafluorobenzohydrazide (compound **26**), N- {[(2-bromo-4-nitrophenyl)amino]carbonothioyl}-3-(2-thienyl)acrylamide (compound **48**), 4-{4-[(2,4-dinitrophenyl)amino]phenyl}amino)-4-oxo-2-butenoic acid (compound **98**) were from ChemBridge Corporation. (S)-(+)-4-benzyl-3-crotonyl-2-oxazolidinone (locostatin) used for NMR analysis was from Aldrich. Protease inhibitor cocktails and locostatin unless otherwise indicated were from Calbiochem. All cell culture media and supplements were from GIBCO (Invitrogen). ^15^N-ammonium chloride was from Cambridge Isotope Laboratories. Anti-phospho and -total ERK antibodies were from Cell Signaling Technology. Anti-rabbit and –mouse IR dye secondary antibodies as well as Odyssey blocking buffer used for Western blotting were purchased from LI-COR Biosciences. Anti-RKIP antibody was developed as described [3]. IgG sepharose beads were from Amersham Biosciences (GE Healthcare). Biotinylated thrombin (50 U) was from Novagen.

### Cell lines and cell culture

HeLa cells stably expressing short hairpin RNA vectors for depleting human RKIP or rat RKIP control (wild-type RKIP) were maintained at 37°C/5% CO_2_ in Dulbecco's modified Eagle's medium (DMEM) supplemented with 10% fetal bovine serum, 50 U/ml penicillin and 50 µg/ml streptomycin under puromycin (2 µg/ml) selection as previously described [1].

### Protein expression

BL21(DE3) cells (Novagen) [31] harboring a pMCSG7-derived expression vector [32] for rat RKIP were grown at 37°C in the M9 media with^15^N-NH_3_Cl as the sole nitrogen source and supplemented with ampicillin (100 µg/ml). The expression construct consisted of a His_6_ tag fused in frame with a TEV protease cleavage signal and the entire open reading frame (residues 1-187) of wild-type rat RKIP (rRKIP). Protein expression was induced with 0.2 mM isopropyl-1-thio-β-d-galactoside (IPTG) when the optical density at 600 nm of cultures reached ∼0.7, and expression was allowed to proceed for 16 hours at 30°C. The His-tagged rRKIP expressing cells were harvested by centrifugation and stored at −80°C.

### Protein purification

Cells having expressed His-tagged rRKIP were lysed in 50 mM Tris-HCl pH 8.0 by a combination of hen egg lysozyme (0.5 mg/ml) (Sigma) and sonication. The lysate was cleared by centrifugation. Concentrated NaCl was added to the lysate at a final concentration of 0.5 M. The solution was loaded onto a Ni-affinity column (Ni Sepharose 6 Fast Flow; Amersham) equilibrated with Column Wash Buffer (50 mM Tris-HCl pH 8.0, 500 mM NaCl). The column was washed with 10 column volumes of Column Wash Buffer followed by 5 column volumes of Column Wash Buffer containing 20 mM imidazole. His-rRKIP was eluted from the column with Column Wash Buffer containing 500 mM imidazole. Fractions containing His-rRKIP were pooled, dialyzed into 50 mM Tris-HCl pH 8.0, 250 mM NaCl, 1 mM DTT and cleaved overnight with his-tagged TEV protease to remove the N-terminal His_6_ tag by the protocol previously described [33]. The cleaved protein was purified from the protease and the cleaved affinity tag by passing the cleavage product over the nickel-affinity column and collecting the column flow-through containing the purified protein. Purity was found to be >95% by SDS-Page analysis with yields routinely exceeding 50 mg/L of culture.

Samples for NMR analysis of ligand binding contained 75 µM ^15^N-rRKIP in 50 mM Tris-HCl pH 7.4 containing 100 mM NaCl and 7% D_2_O. Ligand stocks were prepared in the same buffer and the pH value adjusted appropriately.

### NMR Spectroscopy

^1^H,^15^N-HSQC spectra [34] of ^15^N-rRKIP in the presence of the various ligands were acquired for 40 min at 30°C on a Varian Inova 600 spectrometer equipped with a cryogenic probe and a 9-position sample changer. NMR spectra were processed using the NMRPipe suite [35]. Spectra were analyzed and peak list for apo and ligand bound samples were generated using NMRView [36]. Exact peak positions and intensities were obtained using 2-dimensional deconvolution of peak positions with the pkfit program. Global fitting analysis of peak position and intensity was performed using the program GLOVE (pkfit and GLOVE were developed by the Peter E. Wright laboratory, The Scripps Institute) modified to fit the data to a single-site binding model. Errors were estimated through 500 Monte Carlo simulations of the globally fitted data. The residue specific amide proton pKa values were determined by fitting pH dependent changes of the amide ^1^H chemical shifts to the Henderson-Hasselbach equation using SigmaPlot 8.0 (Jandel Scientific).

Compound screening was performed in a two-step manner as follows. In the first step, 2 µl each of 30 compounds (dissolved in DMSO at the concentration of 5 mg/ml) were pooled and added to 540 µl of 50 mM Tris-HCl pH 7.4 containing 100 mM NaCl, 7% D_2_O and 50 µM ^15^N-RKIP. The final concentration of each compound (17 µg/ml) corresponds to 40–50 µM. The ^1^H,^15^N-HSQC spectra for RKIP with each pooled compound were collected and compared with that of RKIP with DMSO (negative control). The concentration (10%) of DMSO used in the initial screen did not significantly alter the HSQC spectrum, indicating that RKIP was stable in the presence of 10% DMSO. Hit compounds were identified by individually analyzing each of the compounds in those initial mixtures that gave significant HSQC perturbation.

### In vitro Raf kinase assay

The pRav-Flag-Raf-1 plasmid (TAP-Raf-1; Tandem Affinity Purification using Protein A and Flag tagged-Raf-1) was constructed as previously described [21] and stably expressed in H19-7 cells. Briefly, TAP-Raf-1 was immunoprecipitated from H19-7 cells that were serum starved overnight in DMEM media with no supplements, and TAP-Raf was isolated on IgG sepharose beads. Cells were lysed in TAP-lysis buffer (10 mM HEPES pH 7.4, 3 mM MgCl_2_, 10 mM KCl, 5% Glycerol, 0.1% NP40) and cleared by centrifugation. Cell lysates were combined with pre-equilibrated TAP-lysis buffer IgG sepharose beads and incubated for 1 h at 4°C. Beads were washed with TAP-lysis buffer and equilibrated with binding buffer (10 mM HEPES pH 7.4, 3 mM MgCl_2_, 10 mM KCl, 150 mM NaCl). Beads were aliquoted and combined with RKIP (5 µg) and increasing concentrations of RKIP binding compounds (**98**, **26** or **48**), locostatin or 1,2-dihexanoyl-sn-glycero-3-phosphoethanolamine (DHPE) and incubated for 30 min at 4°C. Complexes were washed three times with binding buffer with corresponding concentrations of RKIP binding compounds (**98**, **26** or **48**) or locostatin or DHPE and boiled in sample buffer (2X). Lysates were separated on SDS-PAGE (12%), transferred to nitrocellulose and immunoblotted with anti-RKIP antibody.

### RKIP phosphorylation

*In vitro* phosphorylation of RKIP by PKCα was as previously described [21]. Briefly, RKIP (5 µg) was combined with increasing concentrations of RKIP binding compounds (**98**, **26** or **48**), locostatin or DHPE and the reaction was initiated with 100 µM ATP containing 5 µCi of [γ-^32^P]ATP (Perkin Elmer) at 30°C for 10 min. The reaction was terminated using sample buffer (6X) and boiling at 100°C for 5 min. Lysates were separated on SDS-PAGE (12%), transferred to nitrocellulose and visualized by exposing to film. Results were quantified using phospho-imager screens (Molecular Dynamics) and analyzed with ImageQuant software [3].

### MAPK activation assay

HeLa cells (wild-type or RKIP depleted) were seeded in 6-well plates at 0.5×10^6^/ml and allowed to adhere overnight at 37°C [1]. Cells were serum starved overnight in DMEM media with no supplements. The next day, cells were treated with 0.1% DMSO (vehicle control), 20 µM locostatin or RKIP binding compounds (at indicated concentrations) for 25 min followed by EGF (10 ng/ml) for 5 min at 37°C. The reaction was terminated by placing plates on ice and washing with cold PBS (containing protease inhibitors). Cells were homogenized by cell scraping in RIPA lysis buffer supplemented with sodium orthovanadate (0.1 mM), sodium fluoride (0.5 mM) and protease inhibitor mixture tablet. Cell lysates were sheared five times in a 1 ml tuberculin syringe attached with a 21 G ×19 mm needle, left on ice for 1 h and then spun at 16, 000×*g* for 15 min at 4°C. Cell lysates (30 µg) were resolved by SDS-PAGE (12.5%), transferred to nitrocellulose and analysed by Western blotting with anti-phospho ERK or anti-total ERK (Cell Signaling Technology, Inc) primary antibodies. Membranes were probed with anti-rabbit or –mouse IR dye secondary antibodies (LI-COR Biosciences). The amount of MAPK activity determined for each sample was normalized to total ERK in each sample. Digital analysis of immunoreactivity was done using LI-COR Biosciences Infrared Imaging System which is quantitative and independent of exposure time. Odyssey software (version 2.1; Lincoln, NE) was used for analysis of the immunoblots.

### Cell Viability assay

Cell viability was determined as a measure of metabolic activity according to manufacturer's protocol with minor modifications. Briefly, HeLa cells (wild-type or RKIP depleted) were seeded in 96-well plates at 4×10^4^/ml and allowed to adhere overnight at 37°C. The next day, cells were treated with 0.1% DMSO (vehicle control) or 20 µM each of locostatin or RKIP binding compounds for 30 min followed by 20 µl cell titer blue reagent resazurin (Promega) for 2 h at 37°C. Metabolic activity was measured using a fluorometer at 560_Ex_/590_Em._

### Statistical analysis

Differences between compound treated or untreated cells were compared using a two-tailed unpaired Student's t-test.

## Supporting Information

Figure S1 Overlay of 1H, 15N-HSQC spectra of RKIP in the absence (black) and presence (red) of a potential ligand that our assay did not detect significant binding. The spectra are presented in the same manner as in Fig. 1. Compounds tested are: (A) cAMP (5 mM), (B) GTP (130 mM), (C) O-phosphorylserine (5 mM), (D) O-phosphoryltyrosine (5 mM), (E) platelet activating factor (13.8 mM). Their chemical structures are shown in Table 1. A small number of cross peaks are affected in these spectra, but the perturbations are due to a small change in the pH (less than 0.1 pH unit). This conclusion is confirmed by the spectrum (F), in which the same set of peaks is affected by a small pH shift. Cross peaks that are particularly sensitive to pH changes are enclosed in blue boxes.
(0.83 MB TIF)

Table S1 Summary of the NMR-based binding assays for potential RKIP ligands.
(0.22 MB DOC)
