# Supplementary figures and images for: Characterization of the Raf Kinase Inhibitory Protein (RKIP) Binding Pocket: NMR-Based Screening Identifies Small-Molecule Ligands
Source: PLoS One. 2010 May 5;5(5):e10479. doi: 10.1371/journal.pone.0010479 (PMC2864760; doi:10.1371/journal.pone.0010479)

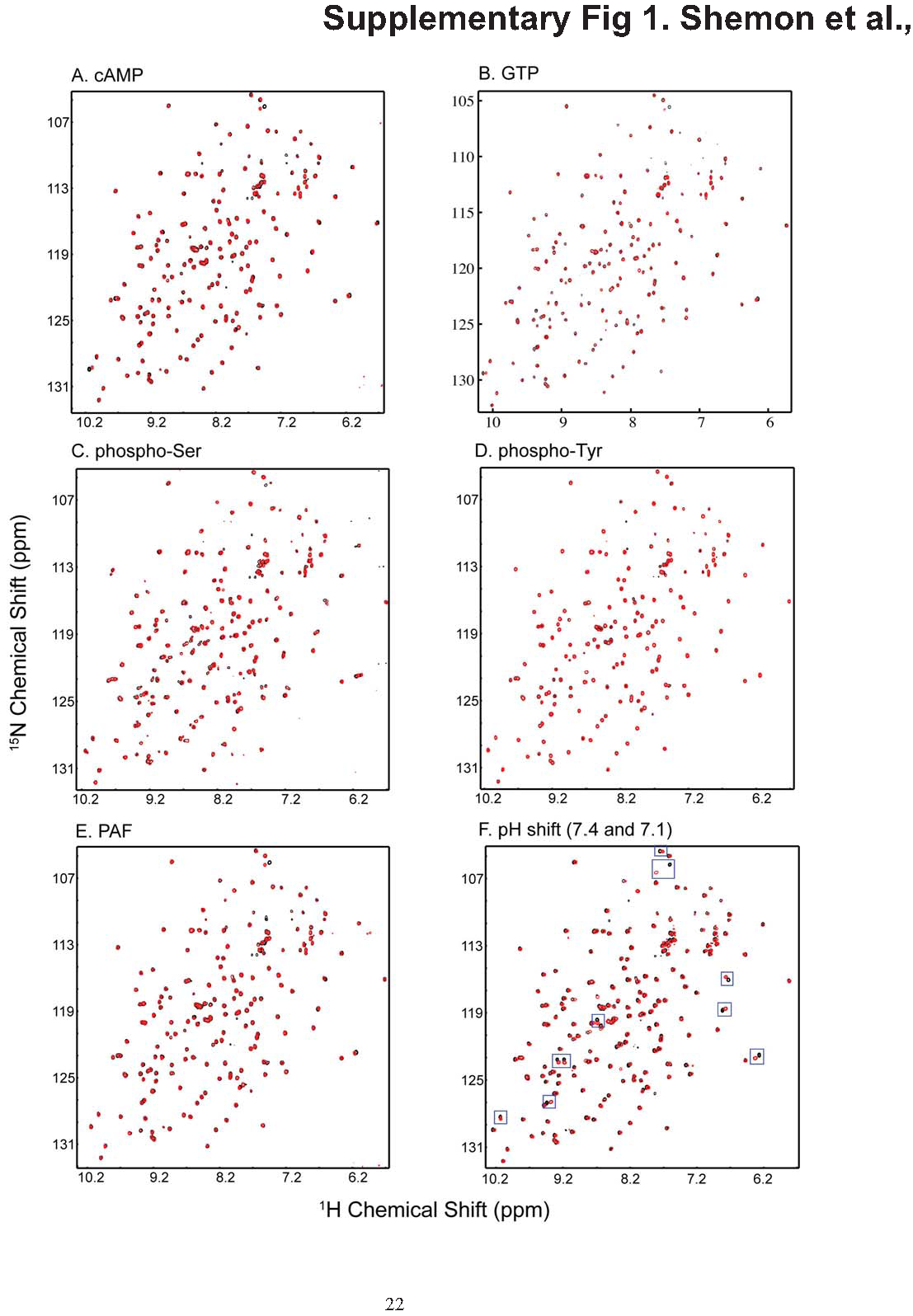

Supplement: Figure S1 — Overlay of 1H, 15N-HSQC spectra of RKIP in the absence (black) and presence (red) of a potential ligand that our assay did not detect significant binding. The spectra are presented in the same manner as in Fig. 1. Compounds tested are: (A) cAMP (5 mM), (B) GTP (130 mM), (C) O-phosphorylserine (5 mM), (D) O-phosphoryltyrosine (5 mM), (E) platelet activating factor (13.8 mM). Their chemical structures are shown in Table 1. A small number of cross peaks are affected in these spectra, but the perturbations are due to a small change in the pH (less than 0.1 pH unit). This conclusion is confirmed by the spectrum (F), in which the same set of peaks is affected by a small pH shift. Cross peaks that are particularly sensitive to pH changes are enclosed in blue boxes. (0.83 MB TIF) [file pone.0010479.s001.tif]
