# Supplementary material for: Characterization of the Raf Kinase Inhibitory Protein (RKIP) Binding Pocket: NMR-Based Screening Identifies Small-Molecule Ligands
Source: PLoS One. 2010 May 5;5(5):e10479. doi: 10.1371/journal.pone.0010479 (PMC2864760; doi:10.1371/journal.pone.0010479)
Supplement: Table S1 — Summary of the NMR-based binding assays for potential RKIP ligands. (0.22 MB DOC) [file pone.0010479.s002.doc]

|  |  | | *K*_d_ (mM) | | | *K*_d_ (mM) | | Maximum conc. | |  |
| --- | --- | --- | --- | --- | --- | --- | --- | --- | --- | --- |
| Structure | | Compound | pH 7.4 | | pH 6.0 | | | assayed (mM) | |  |
| 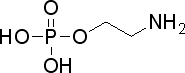 | O-phosphorylethanolamine | | | nb^a^ | | | weak^a^ | | 100 | |
| 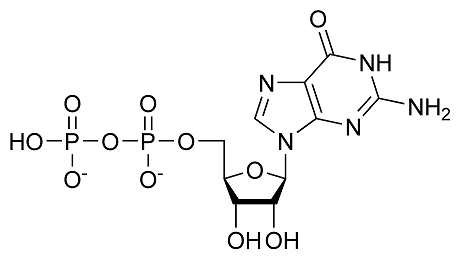 | GDP | | | nb | | | - | | 130 | |
| 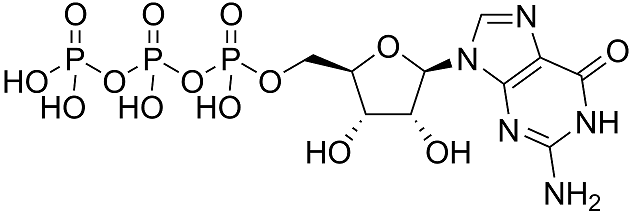 | GTP | | | nb | | | - | | 130 | |
| 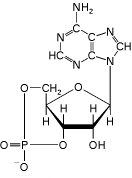 | cAMP | | | nb | | | - | | 5 | |
|  | Inorganic phosphate (P*_i_*) | | | nb | | | - | | 130 | |
| 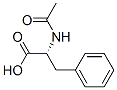 | Phenylalanine | | | nb | | | - | | 29 | |
| 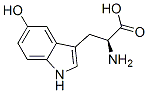 | Tryptophan | | | nb | | | - | | 10 | |
| 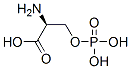 | O-phosphorylserine | | | nb | | | nb | | 5 | |
| 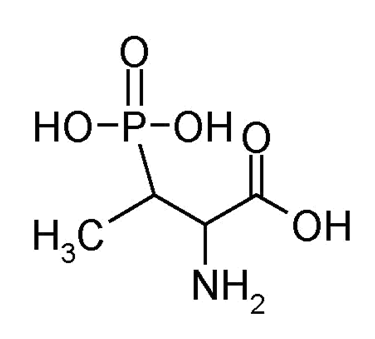 | O-phosphorylthreonine | | | nb | | | nb | | 5 | |
| 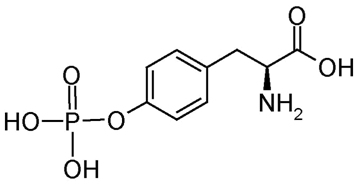 | O-phosphoryltyrosine | | | nb | | | weak | | 5 | |
| 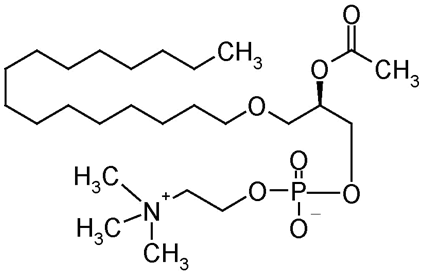 | Platelet Activating Factor | | | nb | | | - | | 13.8 | |
| 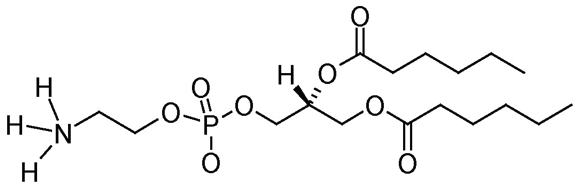 | DHPE | | | 1.15 ± 0.05 | | | nd | | 4.5 | |
| 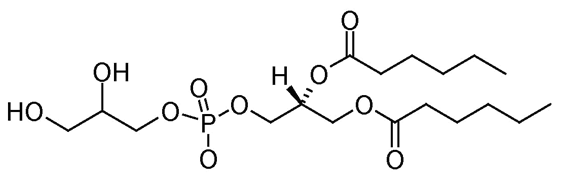 | DHPG | | | 0.94 ± 0.04 | | | 1.21 ± 0.07 | | 4.5 | |
| 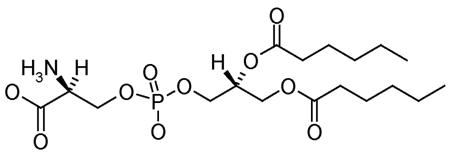 | DHPS | | | 0.95 ± 0.04 | | | 1.50 ± 0.09 | | 4.5 | |
| 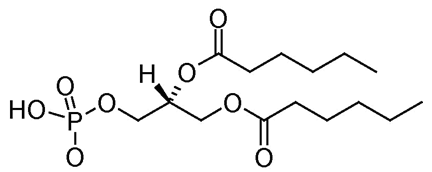 | PA | | | 1.36 ± 0.07 | | | 0.30 ± 0.01 | | 4.5 | |

^a^ nb, no binding detected; weak, weak binding may exist but the *K*_d_ could not be determined; nd, not determined
